# The role of perioperative factors in the prognosis of cancer patients: A coin has two sides

**DOI:** 10.7555/JBR.38.20240164

**Published:** 2024-09-24

**Authors:** Yingzhou Tu, Sen Wang, Haoran Wang, Peiyao Zhang, Mengyu Wang, Cunming Liu, Chun Yang, Riyue Jiang

**Affiliations:** 1 Department of Anesthesiology and Perioperative Medicine, the First Affiliated Hospital of Nanjing Medical University, Nanjing, Jiangsu 210029, China; 2 Department of Radiation Oncology, the First Affiliated Hospital of Nanjing Medical University, Nanjing, Jiangsu 210029, China

**Keywords:** cancer recurrence, anesthesia medications, anesthetic techniques, perioperative period, surgery

## Abstract

Cancer, potentially the second leading cause of mortality globally, poses a significant health challenge. The conventional treatment for solid tumors typically involves surgical intervention, followed by chemotherapy, radiotherapy, and targeted therapies. However, cancer recurrence and metastasis remain major issues. Anesthesia is essential for ensuring patient comfort and safety during surgery. Despite its crucial role in surgery, the precise effect of anesthesia on cancer patients' outcomes has not been clearly understood. This comprehensive review aims to elucidate perioperative anesthesia strategies for cancer patients and their potential effects on prognosis. Given the complexity of cancer treatments, understanding the relationship between anesthesia and cancer outcomes is crucial. By examining potential implications of anesthesia strategies for cancer prognosis, this review may help better understand treatment efficacy and risk factors for cancer recurrence and metastasis. Ultimately, a detailed analysis of anesthesia practices in cancer surgery may provide insights to refine existing anesthesia protocols and reduce risk factors for poor patient outcomes.

## Introduction

Cancer morbidity and mortality have rapidly escalated globally, with 19.3 million new cancer cases and nearly 10 million cancer-related deaths in 2020, and an estimated 28.4 million new cases by 2040^[1]^. While surgery remains the primary treatment for patients with solid tumors, many patients still succumb to the disease postoperatively because of cancer recurrence and metastasis, even after chemo- and radio-therapies^[2]^. Surgical injury and perioperative stress can impair immune function, potentially contributing to recurrence and metastasis^[3]^. Several studies suggest that certain anesthetic agents, such as volatile anesthetics and opioids, may promote cancer cell survival, angiogenesis, and metastasis^[4]^. Conversely, other research indicates that regional anesthesia techniques, like epidurals, may reduce the risk of cancer recurrence^[5]^. This reduction may be attributed to the preservation of immune function and a decreased need for systemic opioids. However, the lack of large-scale, randomized controlled trials complicates the issue. Consequently, the effect of perioperative anesthesia-related factors on the prognosis of cancer patients remains inconclusive. Therefore, we summarized the effects of perioperative anesthesia, including analgesics, anesthesia methods, and other perioperative anesthesia factors, on cancer patient prognosis.

## Perioperative anesthesia and analgesic medications

### Intravenous anesthetics

#### Propofol

Propofol, a commonly used intravenous anesthetic in clinical practice, is often used for anesthesia induction and maintenance. In recent years, propofol has been found to possess not only sedative-hypnotic effects but also non-anesthetic properties, such as antitumor activity^[6]^. Numerous studies have reported that propofol inhibits the proliferation and metastasis of various cancer cells, including cancers of the colon, breast, lung, liver, cervix, and esophagus^[7–12]^. Propofol's inhibitory effects on cancer cell growth, migration, invasion, and glycolysis are mediated through diverse signaling pathways. Studies have highlighted its efficacy in suppressing cervical cancer cell viability, colony formation, invasion, and migration, while promoting apoptosis through the HOTAIR/miR129-5p/RPL14 axis^[9]^. Zhao *et al*^[13]^ reported that propofol down-regulated the expression of *circTADA2A* through the miR-455-3p/FOXM1 axis, inhibiting cell carcinogenesis and aerobic glycolysis in lung cancer both *in vitro* and *in vivo*.

In addition to its direct effects on the biological processes of cancer cells, propofol may also affect the immune function of cancer patients after surgery. Studies have demonstrated that propofol enhances the activity of cytotoxic T cells and decreases the production of pro-inflammatory cytokines^[14–15]^. Furthermore, propofol has been shown to enhance the function of peripheral blood natural killer (NK) cells in patients with esophageal squamous cell carcinoma, suggesting its potential to ameliorate postoperative immunosuppression^[16]^.

Propofol has also been found to significantly affect the sensitivity of cancer cells to chemotherapy, thereby influencing the prognosis of cancer patients. For example, propofol enhances the efficacy of paclitaxel by inhibiting the expression of transcription factor SLUG^[17]^. Han *et al*^[18]^ demonstrated that propofol reduced cisplatin resistance in non-small cell lung cancer by inducing ferroptosis, thereby increasing the activity of chemotherapeutic agents.

These findings suggest that propofol plays a multifaceted role in modulating the sensitivity of cancer cells to chemotherapy, with potential implications for improving cancer treatment outcomes.

#### Ketamine

Ketamine, a racemic mixture consisting of (*S*)- and (*R*)-ketamine, is a non-competitive N-methyl-D-aspartate receptor antagonist. In addition to propofol, ketamine has been shown to affect cancer migration and invasion. It may inhibit the expression of vascular endothelial growth factor and reduce cell migration and aerobic glycolysis in colorectal cancer cells, thereby inhibiting cancer progression^[19–20]^. In lung cancer cells, ketamine induces apoptosis by activating the *CD69* gene transcription^[21]^. However, in pancreatic cancer cells, ketamine directly inhibits cancer cell proliferation while also inhibiting apoptosis^[22]^. In contrast, ketamine can exacerbate cancer progression by upregulating levels of the anti-apoptotic protein BCL-2, promoting the invasion and proliferation of breast cancer cells^[23]^. Moreover, Ketamine has been shown to induce lymphocyte apoptosis through the mitochondrial pathway and inhibit the functional maturation of dendritic cells^[24]^. At present, the effect of ketamine on cancer cells remains controversial, and further research is needed to clarify its precise role in cancer progression and treatment.

### Inhalational anesthetics

Inhalation anesthetics, such as sevoflurane, isoflurane, and desflurane, play important roles in clinical anesthesia. Several *in vitro* studies have demonstrated the effects of these inhaled anesthetics on the immune system^[25]^. Various immune cells play antitumor roles in the perioperative period, such as neutrophils, NK cells, B lymphocytes, and T lymphocytes, with NK cells and T lymphocytes being particularly prominent in exerting antitumor effects after surgery^[26–28]^. For innate immunity, several studies have observed that inhaled anesthetics impair neutrophil function^[29]^. As early as 1997, sevoflurane was found to reduce the number of neutrophils^[30]^. At the same time, isoflurane, sevoflurane, and halothane have all been shown to reduce the cytotoxicity of NK cells^[25]^. Combined *in vivo* and *in vitro* studies showed that both the interferon-α and interferon-β induced stimulation of NK cell cytotoxicity was inhibited after exposure to halothane and isoflurane^[31]^. For adaptive immunity, sevoflurane, isoflurane, and desflurane have been found to induce T lymphocyte apoptosis both *in vitro* and *in vivo*. These anesthetics also upregulate hypoxia-inducible factor 1 alpha (HIF-1α) expression to promote angiogenesis, cell proliferation, and metastasis^[32–35]^. In addition, inhalational anesthetics may also affect the neuroendocrine response of the hypothalamic-pituitary-adrenal axis and the sympathetic nervous system, leading to increased secretion of immunosuppressive-related cytokines, such as vascular endothelial growth factor (VEGF) and transforming growth factor β (TGF-β). This occurs through immunomodulatory hormones like catecholamines and prostaglandins, thereby indirectly affecting the immune response^[36]^.

### Analgesics

#### Opioid analgesics

Opioids play a crucial role in providing postoperative analgesia for cancer patients. However, their potential effect on cancer recurrence and metastasis remains largely unknown. Studies have revealed that opioids disrupt the functions of immune cells. For instance, fentanyl has been shown to inhibit the activity of macrophages and NK cells, while morphine is known to reversibly inhibit the cytotoxic effects of NK cells and the activation of cytotoxic T lymphocytes, particularly CD4^+^CD8^+^ cells^[37–39]^. Additionally, both *in vitro* and *in vivo* studies have shown that morphine at clinically relevant doses promotes angiogenesis, thereby facilitating the nutrient supply and growth of tumors, and aiding in the evasion of host defenses^[40]^. Morphine has also been correlated with promoting lymphangiogenesis, as well as mast cell activation and degranulation, which leads to the increased levels of inflammatory cytokines^[41]^.

In addition to numerous cell and animal studies, the effects of opioids on the prognosis of cancer patients have also been investigated in several clinical trials. Some studies have shown that higher intraoperative opioid doses are associated with a decreased risk of cancer recurrence in patients undergoing surgery for stage Ⅰ–Ⅲ adenocarcinoma of the colon^[42]^. However, Sessler *et al*^[43]^ reported that perioperative opioid use was not associated with an increased risk of breast cancer recurrence. Notably, a recent study reported that intraoperative opioids were even protective against the recurrence of triple-negative breast cancer^[44]^. Furthermore, a randomized prospective clinical trial found that opioid use did not significantly affect biochemical recurrence rates or biochemical recurrence-free survival in post-prostatectomy patients at moderate or high risk of biochemical recurrence^[45]^. Therefore, the effects of perioperative opioid use on the prognosis of cancer patients remain inconclusive, which requires future investigations.

#### Non-opioid analgesics

Non-opioid analgesics commonly used perioperatively include non-selective cyclooxygenase (COX) inhibitors and selective COX-2 inhibitors. Numerous studies have shown that non-selective COX inhibitors, such as non-steroidal anti-inflammatory drugs, reduce the risk and mortality rate in patients with cancer^[46]^. COX-2 is widely expressed in various cancers, playing a crucial role in carcinogenesis, cancer progression, and resistance to chemotherapy and radiotherapy^[47]^. Non-steroidal anti-inflammatory drugs inhibit COX activity, blocking the conversion of arachidonic acid to prostaglandins, and indirectly affecting cancer progression and metastasis through their analgesic effects, potentially benefiting patients. Similarly, selective COX-2 inhibitors may affect the long-term prognosis of cancer patients. Interestingly, research indicates that these inhibitors help control chronic postoperative pain in esophageal cancer patients and may prolong patient survival^[48]^. Celecoxib has been shown to reduce the incidence of colorectal adenomas and colorectal cancer^[49]^. However, a recent randomized clinical trial involving 2526 patients with stage Ⅲ colon cancer found that the addition of celecoxib to standard adjuvant chemotherapy did not significantly improve disease-free survival (DFS), compared with placebo^[50]^. In conclusion, future large-scale patient cohort studies are needed to further investigate the effects of non-opioid analgesics on cancer prognosis.

### Local anesthetics

Lidocaine, a widely used amide local anesthetic, is commonly administrated *via* systemic intravenous infusion or nerve blocks. It acts on cancer cells and the tumor microenvironment both *in vivo* and *in vitro* in ways similar to other anesthetics. Lidocaine may enhance the function of NK cells by modulating the release of lytic granules, essential components of the antitumor immune response^[51]^. Moreover, perioperative use of lidocaine has been found to inhibit immune cell infiltration into the premetastatic microenvironment and prevent the release of pro-metastatic inflammatory cytokines, thereby reducing the risk of metastases^[52]^. Lidocaine may also enhance the efficacy of traditional chemotherapy drugs. For example, Xing *et al*^[53]^ demonstrated that lidocaine inhibited tumor growth and increased tumor sensitivity to cisplatin treatment in a xenograft model. Lidocaine not only inhibits cancer progression but also alleviates pain, reduces surgical irritation, and mitigates stress responses in various cancers. By effectively reducing postoperative pain and the inflammatory response in cancer^[54]^, lidocaine may serve as a valuable adjuvant drug for cancer treatment.

Recent clinical studies have investigated potential benefits of intraoperative lidocaine use in improving long-term survival of cancer patients. A study on clinical efficacy of lidocaine in the treatment of pancreatic cancer recurrence found that patients treated with intravenous lidocaine had better survival rates at one and three years, although there was no significant difference in DFS^[55]^. However, a multicenter randomized controlled trial found that intraoperative lidocaine infusion did not improve overall survival (OS) or DFS in patients undergoing pancreatectomy for pancreatic cancer; notably, it did reduce the formation of circulating neutrophil extracellular traps, which are associated with a poor prognosis in pancreatic tumor tissues^[56]^. Similarly, intraoperative intravenous lidocaine infusion was correlated with an improved OS or DFS in patients undergoing primary cytoreductive surgery of ovarian cancer, as well as a reduction in intraoperative opioid use^[57]^. Furthermore, a prospective randomized controlled trial on ovarian tumor resection found that intraoperative and 72-hour intraperitoneal injections of ropivacaine shortened the time to chemotherapy administration^[58]^. Despite these promising findings, the use of lidocaine as an adjuvant therapy requires further investigation through large-scale, scientifically rigorous prospective and randomized controlled trials to establish its safety and efficacy conclusively.

## Anesthetic techniques

### General anesthesia

General anesthesia plays a critical role in cancer surgery, with the two main methods being inhalation anesthesia based on volatile anesthetics and total intravenous anesthesia using propofol. Emerging evidence indicates a potential association between volatile anesthetic-based inhalation anesthesia and a worse long-term cancer prognosis, compared with propofol-based intravenous anesthesia. Preclinical studies also revealed that anesthetics affected cellular immunity and influenced cancer cell proliferation, migration, and invasion^[59]^. Notably, inhalation anesthetics may directly inhibit both innate and adaptive immunity, leading to reduced neutrophil recruitment and adhesion, decreased macrophage phagocytosis, impaired cytotoxicity of NK cells, and polarization of T lymphocytes to tumorigenic Th2 cell populations^[17]^. In contrast, intravenous anesthetics, such as propofol, have been shown to positively modulate immune function in cancer patients, thereby exhibiting some antitumor effects^[60]^.

Current clinical studies mainly focus on comparing the effects and outcomes of inhalation anesthesia versus intravenous anesthesia in cancer patients undergoing surgery. Seo *et al*^[61]^ reported that patients undergoing surgery for non-small cell lung cancer exhibited a more than 20% higher recurrence risk in the inhalation anesthesia group, compared with the intravenous anesthesia group. Similarly, a large study including colon cancer patients demonstrated better survival outcomes with intravenous anesthesia alone across different tumor-node-metastasis stages^[62]^. However, conflicting results have also been reported in patients undergoing breast cancer surgery, with some studies suggesting a lower risk of cancer recurrence with the propofol-based intravenous anesthesia, while others show no significant difference in postoperative survival outcomes between the two methods^[14,63]^. A long-term follow-up of a multicenter randomized trial, which enrolled 1228 patients aged 65 to 90 years scheduled for major cancer surgery, found no difference between propofol-based intravenous anesthesia and sevoflurane-based inhalational anesthesia in terms of recurrence-free survival and event-free survival^[64]^. Similarly, a retrospective cohort study investigating the effects of anesthesia type on survival outcomes in patients undergoing elective surgical resection for papillary thyroid carcinoma found that propofol anesthesia was not associated with a better survival, compared with desflurane anesthesia^[65]^. Another systematic review and meta-analysis, however, highlighted the potential for improved OS during cancer surgery with the propofol-based intravenous anesthesia, compared with inhaled anesthesia^[66]^. Those conflicting findings underscore the need for more prospective multicenter studies with larger sample sizes to comprehensively investigate the effects of these two main anesthesia modalities on cancer recurrence related to surgical procedures.

### Regional anesthesia

Regional anesthesia techniques, such as epidural anesthesia, spinal anesthesia, and nerve blocks, temporarily block the conduction function of the spinal cord or peripheral nerves to achieve anesthesia and analgesia. It has been suggested that regional anesthesia may reduce surgical stress response and immunosuppression, decrease the need for volatile anesthesia, and reduce pain and opioid requirements, potentially mitigating perioperative tumor pathways and improving long-term oncological outcomes^[67]^. A randomized controlled trial found a significant reduction in both NK cell and T cell activities in patients who received regional anesthesia, compared with those who received general anesthesia^[68]^.

However, because of the heterogeneity of surgical scope, cancer type, patient characteristics, and limitations of study types, clinical studies on the long-term prognosis of cancer patients have yielded inconsistent results. Many studies compared regional anesthesia with general anesthesia alone instead of their combined effects. For example, Xu *et al*^[69]^ investigated the outcomes of adult patients undergoing thoracoscopic lung cancer resection and found that epidural analgesia combined with a thoracic epidural block did not improve recurrence-free survival or other survival outcomes, nor did it benefit patients diagnosed with lung cancer. Likewise, in a randomized controlled trial of elderly patients undergoing major thoracic and abdominal surgery, general anesthesia combined with thoracic epidural block and epidural analgesia did not enhance OS or tumor-specific survival, although it did reduce the use of inhaled anesthetics and long-acting opioids^[70]^.

Conversely, studies examining the effect of regional analgesia on breast cancer recurrence have suggested that a regional block combined with general anesthesia may be an appropriate anesthetic strategy for patients with breast cancer. For example, one case-control study revealed that paravertebral block-regional anesthesia with the propofol-based sedation reduced locoregional recurrence in breast cancer patients undergoing breast-conserving surgery^[71]^. Additionally, a retrospective study found that patients with non-muscle-invasive bladder cancer who underwent transurethral resection under neuraxial anesthesia had a significantly longer recurrence-free survival, compared with those who received general anesthesia^[72]^.

In conclusion, current clinical trials do not offer any unequivocal evidence that regional anesthesia improves the long-term prognosis of cancer patients. Therefore, the decision to use regional anesthesia perioperatively should be based on patient characteristics and specific surgical needs, rather than specifically on its potential to prevent cancer recurrence.

## Other anesthesia-related perioperative factors

### Blood pressure

Hypotension during the perioperative period may lead to an inadequate perfusion of vital organs, potentially causing acute or chronic irreversible damage and significantly affecting the postoperative outcomes. Notably, perioperative blood pressure is not only associated with adverse cardiovascular events and organ damage but also affects short- and long-term mortality. Moreover, emerging evidence suggests that perioperative blood pressure may impact cancer outcomes. Perioperative hypotension can activate the sympathetic nervous system and the hypothalamic-pituitary-adrenal axis, subsequently diminishing tissue perfusion and leading to cellular hypoxia, hypoxemia, metabolic acidosis, and increased lactate levels in severe cases. This, in turn, may impair immune cell function and promote cancer cell proliferation and metastasis. For example, Younes *et al*^[73]^ observed that the frequency of hypotensive episodes played a pivotal role in influencing cancer recurrence rates in patients who had undergone complete resection of colorectal liver metastases. Consequently, they recommended avoiding hypotensive episodes during surgery to maximize the chance of cure and extend DSF in these patients. Similarly, Park *et al*^[74]^ conducted a retrospective study involving renal cell carcinoma patients and found that perioperative blood pressure in the stage 2 hypertension range (≥ 160/100 mmHg) was an independent predictor of overall mortality. Despite these findings, the potential effect of perioperative blood pressure fluctuations on cancer prognosis warrants further investigation.

### Blood transfusion

Transfusion-related immunomodulation refers to the immunosuppressive effects of allogeneic transfusions in the perioperative period, which may negatively influence cancer recurrence and metastasis. Allogeneic transfusions alter several aspects of the recipient's immune function, including a decreased ratio of helper T lymphocytes to suppressor T lymphocytes, diminished NK cell function, defective antigen presentation, and reduced cell-mediated immunity^[75]^. Furthermore, studies in various experimental animal models have indicated that the pro-tumor growth effects of allogeneic transfusion may be attributed to the presence of allogeneic donor leukocytes in transfused blood products^[76]^.

Although limited evidence from randomized trials exists concerning the association of blood transfusion with cancer recurrence and prognosis, most current relevant clinical studies are retrospective. A propensity-matched analysis of patients with hepatocellular carcinoma undergoing hepatectomy revealed that red blood cell transfusion (RBCT) promoted cancer recurrence and decreased long-term survival after radical resection, while other types of transfusions, including platelets, 5% albumin, and 25% albumin, did not affect long-term survival^[77]^. Similar associations have also been observed in gastric cancer, non-small cell lung cancer, and head and neck cancers^[78–80]^. Moreover, a retrospective single-center cohort study of patients with stage Ⅰ–Ⅲ colorectal cancer who underwent radical surgery between 2005 and 2017 found that perioperative RBCT was significantly associated with a poorer OS^[81]^. Survival outcomes in the RBCT group were significantly worse than in the non-RBCT group, and perioperative RBCT was significantly associated with a poorer OS in multivariable analysis^[81]^. Notably, when propensity scores were used to match transfused and non-transfused cases, no difference in OS and cancer-specific survival was observed^[81]^. The need for intraoperative blood transfusion is influenced by factors such as surgical complexity and the patient's physical condition. Similarly, cancer recurrence and prognosis are influenced by variables such as tumor stage and postoperative adjuvant therapy. Therefore, the effect of blood transfusion on cancer prognosis remains uncertain, highlighting the need for well-designed randomized controlled trials to provide conclusive evidence.

### Hypothermia

Hypothermia is a frequent perioperative complication that may lead to various adverse outcomes including postoperative infection, cardiovascular events, and an increased risk of blood transfusion^[82]^. Elevated temperatures generally promote the activation, function, and delivery of immune cells, while decreased temperatures inhibit these processes^[83]^. In 2020, Zeba *et al*^[84]^ conducted a single-center randomized controlled trial to investigate the effect of intraoperative hypothermia on the cytokine profile. They found that patients in the non-warming group had the lowest mean of the perioperative core body temperature and showed a sustained increase in the pro-inflammatory response. Additionally, intraoperative warming to maintain normal body temperature was found to attenuate the harmful pro-inflammatory response. Furthermore, Nduka *et al*^[85]^ used an animal model to investigate the effect of hypothermia on tumor growth during laparoscopic surgery. Their results showed that the tumor mass was significantly increased in the cold CO_2_ pneumoperitoneum group, compared with the warmed CO_2_ pneumoperitoneum group. However, few clinical studies investigated the association between intraoperative hypothermia and cancer prognosis, and many factors may affect cancer prognosis, making it difficult to explain the differences in long-term outcomes of cancer patients with intraoperative hypothermia alone. A retrospective clinical study of 124 patients with muscle-invasive bladder cancer who underwent radical cystectomy between 2003 and 2016 showed no significant differences in OS and DFS between the hypothermic and normothermic groups^[86]^. However, in subgroup analysis based on pathologic stage, both OS and DFS were significantly shorter in the hypothermic group than in the normothermic group for stage Ⅱ patients^[86]^. Expanding on this, Lyon *et al*^[87]^ increased the sample size to 852 patients undergoing radical cystectomy and found no significant difference in the two-year survival rate between the hypothermic and the normothermic groups, and no significant association between intraoperative hypothermia and recurrence-free survival, tumor-specific survival, and OS. However, they found that a median body temperature lower than 35 ℃ was an independent factor influencing OS.

Given the influence of hypothermia on tumor growth, hyperthermia has emerged as a potential cancer treatment to reduce cancer recurrence. Current evidence suggests that physiological responses to hyperthermia may enhance the ability of the microenvironment to resist tumors by regulating temperature-sensitive checkpoints in tumor vascular perfusion and metabolism^[88]^. However, an *in vitro* study reported that cancer cells were more resistant to higher temperatures than normal cells, signifying a need for further understanding of the effects of thermal stimulation on the tumor environment and antitumor immune responses^[89]^.

## Limitations

This review summarizes the effects of anesthetics on tumor proliferation, apoptosis, and invasion, primarily using *in vitro* studies. However, several limitations must be acknowledged. First, some epidemiological results are inconsistent with findings from the *in vitro* cell studies. This discrepancy may be attributed to the fact that these *in vitro* studies assess the direct effects of anesthetics on tumor cells, whereas, in clinical practice, tumor tissues are typically excised, and anesthetics exert only transient effects on systemic tissues rather than directly affecting the tumor tissue itself. As a result, these *in vitro* findings may not accurately reflect the effects of anesthetics on tumor recurrence or metastasis in clinical settings^[43,64]^. Furthermore, surgical proficiency critically affects surgery duration and tumor removal thoroughness. Incomplete tumor resection, often because of less meticulous surgical techniques, is a well-documented cause of cancer recurrence. Additional factors, such as intraoperative blood loss, the duration of anesthesia, and the presence of tumor stem cells, also significantly affect surgical outcomes and warrant further investigation. Given these considerations, we plan to undertake a more rigorous systematic review in the future, incorporating a broader range of clinical research data.

## Conclusions and perspectives

**Figure 1:**
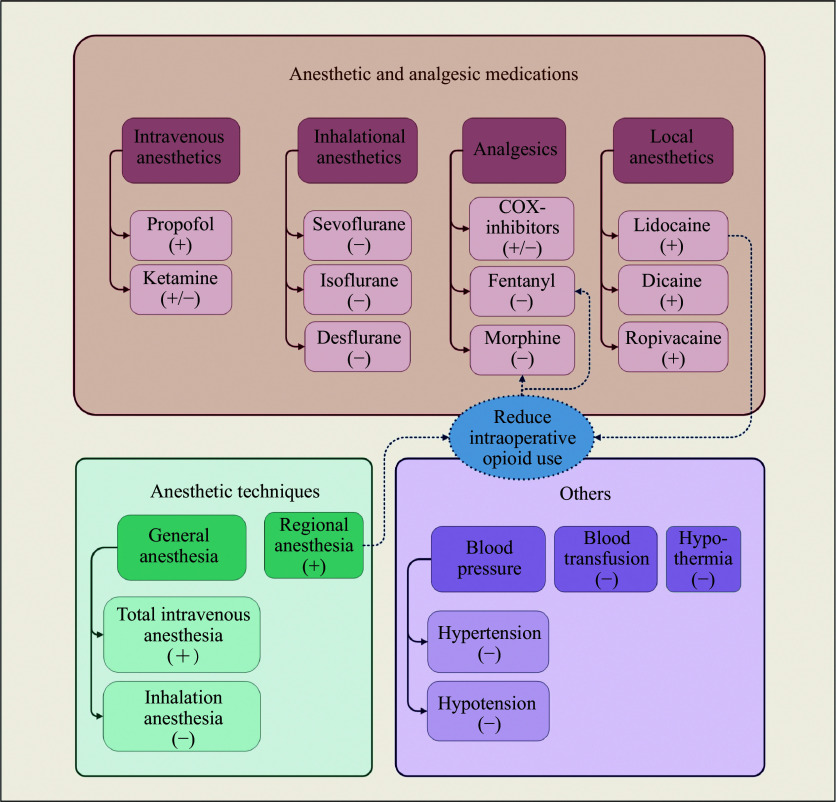
Perioperative factors influencing the prognosis of cancer patients. Abbreviations: (+), positive effects; (−), negative effects; (+/−), controversial effects.

In summary, the immunosuppression caused by anesthetics, surgical techniques, and other perioperative factors may contribute to metastasis and cancer recurrence (***Fig. 1***). However, findings on the effect of anesthesia on immune response and tumor growth remain conflicting (***Table 1***). As a result, the current evidence regarding different anesthetic strategies for perioperative cancer recurrence remains inconclusive. To bridge this knowledge gap, large, randomized multicenter prospective clinical trials are urgently needed to investigate the influence of various anesthetics, surgical techniques, and other relevant factors on the long-term prognosis of cancer surgery.

**Table 1:** Summary of clinical studies assessing the effects of different anesthetic strategies on cancer outcomes

Year	Study type	Group/variable	Cancer	Sample	Outcomes	Reference
2017	Retrospective	TIVA *vs.* inhalation anesthesia	Breast	2729	No difference in RFS.	[63]
2018	Retrospective	Target-controlled infusion with propofol *vs.* desflurane	Colon	1363	Increased in DFS and OS in the propofol group.	[62]
2019	RCT	Regional anesthesia-analgesia (paravertebral blocks and propofol) *vs.* general anesthesia (sevoflurane) and opioid analgesia	Breast	2132	No difference in cancer recurrence.	[43]
2020	Retrospective	Non-lidocaine *vs.* lidocaine	Pancreas	2239	No difference in DFS, but increased in OS in the lidocaine group.	[55]
2020	RCT	Intraperitoneal ropivacaine *vs.* saline	Ovary	58	Decreased in time to initiation of chemotherapy in the intraperitoneal ropivacaine group.	[58]
2021	RCT	Opioid-free anesthesia *vs.* opioid-based anesthesia	Prostate	146	No difference in RFS.	[45]
2021	Retrospective	Opioid	Breast	1143	Decreased in RFS, but no difference in OS.	[44]
2021	Retrospective	Non-lidocaine *vs.* lidocaine	Ovary	702	Increased in DFS and OS in the lidocaine group.	[57]
2021	Systematic review and meta-analysis	TIVA *vs.* inhalation anesthesia	Undivided	31	Increased in OS in the TIVA group, but no difference in RFS.	[66]
2021	RCT	Combined intravenous opioid analgesia-GA *vs.* combined epidural-GA	Lung	400	No difference between RFS and OS.	[69]
2021	RCT	GA *vs.* combined epidural-GA	Undivided	1712	No difference between RFS and OS.	[70]
2021	Retrospective	PB-RA *vs.* INHA-GA	Breast	2769	No difference between OS and DM, but decreased in LRR in the PB-RA group.	[71]
2021	Retrospective	SA *vs*. GA	Bladder	300	Increased in cancer recurrence in the GA group, but no difference in RFS.	[72]
2022	Retrospective	Opioid	Colon	1157	Decreased in cancer recurrence in higher intraoperative opioid dose patients, but no difference in OS.	[42]
2022	RCT	Non-lidocaine *vs.* lidocaine	Pancreas	563	No difference between DFS and OS.	[56]
2023	Retrospective	TIVA *vs.* inhalation anesthesia	Lung	2159	Decreased in cancer recurrence, but increased in OS in the TIVA group.	[61]
2023	RCT	TIVA *vs.* inhalation anesthesia	Undivided	1195	No difference in RFS and OS.	[64]
2024	Retrospective	TIVA *vs.* inhalation anesthesia	Thyroid	1209	No difference in OS.	[65]
Abbreviations: RCT, randomized controlled trial; OS, overall survival; DFS, disease-free survival; DM, distant metastasis; GA, general anesthesia; INHA-GA, sevoflurane-based inhalational general anesthesia; PB-RA, propofol-based paravertebral block-regional anesthesia; RFS, recurrence-free survival; SA, spinal anesthesia; TIVA, propofol-based total intravenous anesthesia.						
